# Microsatellite Instability and Colorectal Cancer, Immunohistochemical and Molecular Evaluation by Using DNA Sequencing: A Single Center Experience

**DOI:** 10.30699/ijp.2021.135222.2481

**Published:** 2021-05-05

**Authors:** Bita Geramizadeh, Farzaneh Bozorg-Ghalati, Firoozeh Jafari, Mitra Mirzai, Zahra Jowkar

**Affiliations:** 1 *Transplant Research Center, Shiraz University of Medical Sciences, Shiraz, Iran*; 2 *Department of Pathology, Medical School of Shiraz University, Shiraz, Iran*; 3 *Department of Molecular Medicine, School of Advanced Medical Sciences and Technologies, Shiraz University of Medical Sciences, Shiraz, Iran*

## Abstract

**Background & Objective:**

Microsatellite instability is common in familial colorectal cancers. It can be tested by the molecular and immunohistochemical methods. There are very few studies which address comparing the clinicopathological characteristics of microsatellite stable (MSS) and microsatellite unstable (MSI) colorectal cancers from Iran. In this study, we aimed to evaluate the clinicopathological and immuno-histochemical findings of MSS and MSI colorectal cancers in our Center as the largest Center of gastrointestinal surgery and oncology in the South of Iran. We also compared the immunohistochemical method vs. molecular study using DNA sequencing.

**Methods:**

For 5 years (2015-2019), 34 patients who underwent operation in the affiliated Hospitals of Shiraz University of Medical Sciences were clinically suspected to microsatellite instability (MSI). The molecular diagnostic tests with DNA sequencing were performed. Clinicopathological and immunohistochemical findings of MSI colorectal cancers were compared with those who were stable.

**Results:**

In the South of Iran**, **MSI colorectal cancers were more common in males. These tumors were more common in the right side with more tendencies to produce mucin with lymphocytic infiltration.

**Conclusion:**

Immunohistochemistry would be a specific method for diagnosis of MSI colorectal cancers but may be associated with high rate of false negative results and of low sensitivity. Therefore, we recommend performing molecular studies by DNA sequencing in those colon cancers with clinical suspicion for MSI and negative immunohistochemical findings.

## Introduction

Colorectal cancer (CRC) is very common in Western countries, and its incidence is being increased in the Eastern Mediterranean countries such as Iran (1, 2).

Sporadic colon cancers are more common and only 5-10% of colorectal cancers are considered hereditary. The most common hereditary colon cancer is hereditary nonpolyposis colorectal cancer (HNPCC) which is secondary to microsatellite instability (MMR) (DNA mismatch repair genes i.e. hMLH1, hMSH2, hMSH6, and hPMS2) (3). Classification of CRC as HNPCC is based on the immunohistochemical study of the resected tissue or evaluation of the germ-line mutation analysis by molecular studies. Molecular studies show inactivation or mutation of MMR genes. Immunohistochemical studies show the presence or absence of protein expression. Microsatellite instability (MSI) occurs by the alteration in the length of simple, repetitive microsatellite sequences throughout the whole genome (4). MSI tumors have different sensitivities to chemotherapy which makes MSI analysis very important in the therapeutic decision and related prognosis. Also, MSI tumors have specific clinicopathological features, such as more occurrences in the proximal colon, more mucin production, and more tumor infiltrating lymphocytes (5).

There is not much information about the prevalence of HNPCC and colon cancers with MSI in Iran (6). 

In this study, we evaluated our experience about the clinicopathological findings including demographic, histologic and immunohistochemical findings of colorectal cancers with microsatellite instability in comparison with microsatellite stable colorectal cancers. In the meantime, we tried to compare immunohistochemical method with molecular methods (DNA sequencing) for the diagnosis of microsatellite instability. 

## Material and Methods

**Tumor Selection**

Pathology samples were extracted from 34 patients who had operation with the diagnosis of CRC at affiliated Hospitals of Shiraz University of Medical Sciences (Shiraz, Iran) from 2015 to 2019. All the tumors were clinically suspected to be microsatellite instable (either because of familial tendency, age <50 years, and second cancer).

**Histopathological Features**

Hematoxylin-stained slides of the specimens were reviewed by two pathologists (BG and FJ). The following characteristics were evaluated: tumor grade (well, moderately, or poorly differentiated), histological subtype (mucinous cancers were those containing more than 50% extracellular mucin, medullary types with heavy infiltration of tumor infiltrating lymphocytes (7)). Tumors were also classified according to TNM (Tumor-node-metastasis) staging systems.

**Immunohistochemistry Method**

The samples were fixed in 10 % formaldehyde solution, dehydrated, and placed in paraffin for histological analyses. Five-micrometer-thick sections were prepared and mounted on poly-L-lysine-coated slides. Immunohistochemistry (IHC) analysis was performed using the following monoclonal antibodies: anti-MLH1, anti-MSH2, anti-MSH6, and anti-PMS2 (Table 1). All antibodies used in this work are listed in Table 1.

**Table 1:** Characteristics of antibodies used in immunohistochemistry

Antibodies	Company	Clone	Dilution
MLH1	Masterdiagnostica	MAD-000726QD-3	Pre-diluted
MSH2	Zytomed	MSG031	Pre-diluted
MSH6	Masterdiagnostica	MAD-000635QD-3	Pre-diluted
PMS2	Masterdiagnostica	MAD-000681QD-3	Pre-diluted

**Fig. 1:**
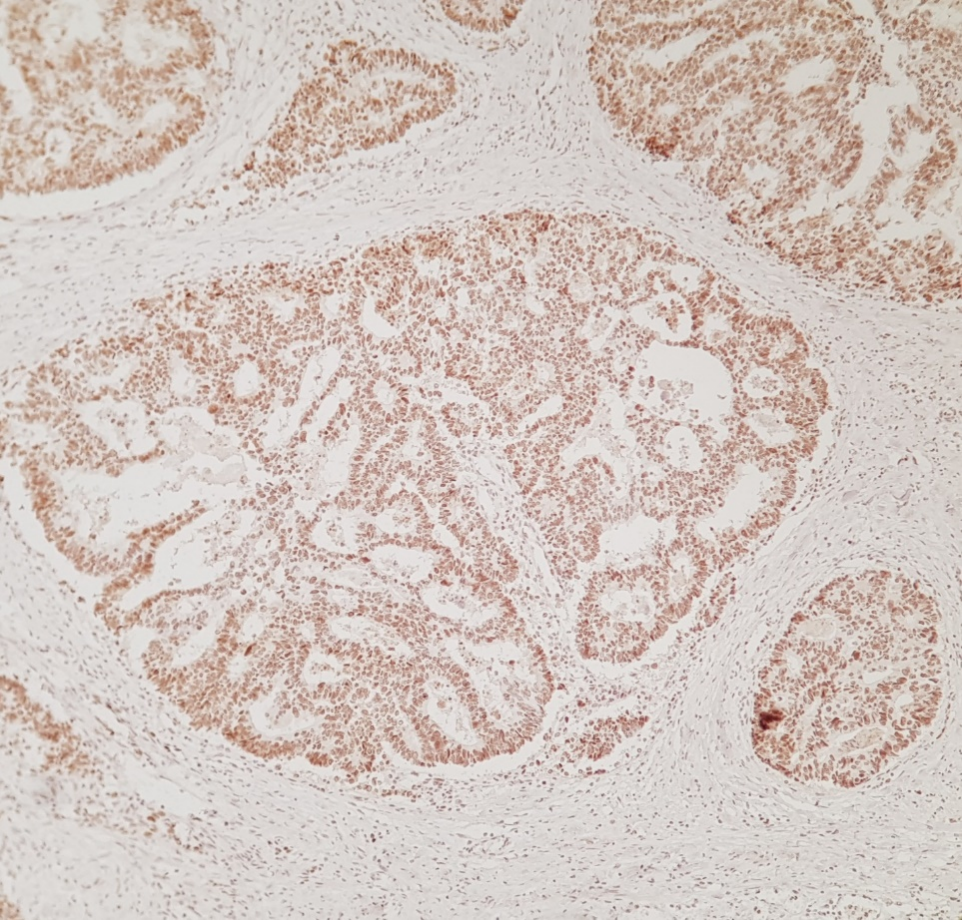
Sections show positive nuclear staining in a case of colorectal adenocarcinoma which means stable and normal MLH 2. (IHC, MLH2 X 250)

Briefly, slides were deparaffinized and endogenous peroxidase activity was blocked by incubation with 3% H_2_O_2_. Heat-induced antigen retrieval was performed using Tris buffer, and the process was performed for 30-60 minutes. After treatment with 10% normal goat serum to block non-specific protein binding, pre-diluted primary antibodies against MLH1, MSH2,MSH6, and PMS2 were applied, followed by incubation with horseradish peroxidase-conjugated multimeric antibody reagent. The antigen-antibody reaction was visualized using diaminobenzidine (DAB) as a chromogen. The slides were counter-stained with hematoxylin. Normal colonic crypt epithelium adjacent to the tumor, lymphocytes and fibroblasts were considered as positive internal control. Loss of expression was recorded when nuclear staining was absent in malignant cells, and present in normal epithelial cells, lymphocytes and fibroblasts. Two observers (BG and FJ) evaluated all cases independently and together. In a few discrepant diagnoses, re-evaluation together solved the issue (Figure 1).

**MSI Analysis by DNA Sequencing**

DNA was extracted from paraffin-embedded tissue samples of primary CRC and paired normal bowel using the DNeasy® tissue kit (Qiagen, Courtaboeuf, France).

Five short tandem repeat (STR) gene markers i.e. NR21, NR24, NR27, BAT25 and BAT26 were analyzed. The related primers were ordered from Macrogene Company (Geum Chun-Gu, Seoul, Korea). These primers were tagged by Hex or Fam fluorescent dyes at 5* sequences (8). The real-time PCR was performed for the target genes in 20 μL reaction volume including 10 μL of 2X SYBR Green master mix without Rox (Ampliqon, Stenhuggervej, Denmark), 50 ng of the extracted DNA, and 1 μL of both reverse and forward primers which were prepared at 10 pM/μL concentration.

The PCR tests were operated in thermal cycler instrument (Rotor-Gene Q, Qiagen, Hilden, Germany). The PCR condition was adjusted at three steps (15 seconds at 95°C, 30 seconds at 60°C, and 60 seconds at 72°C), of 45 cycles followed by melt curve analysis.

**Fig. 2:**
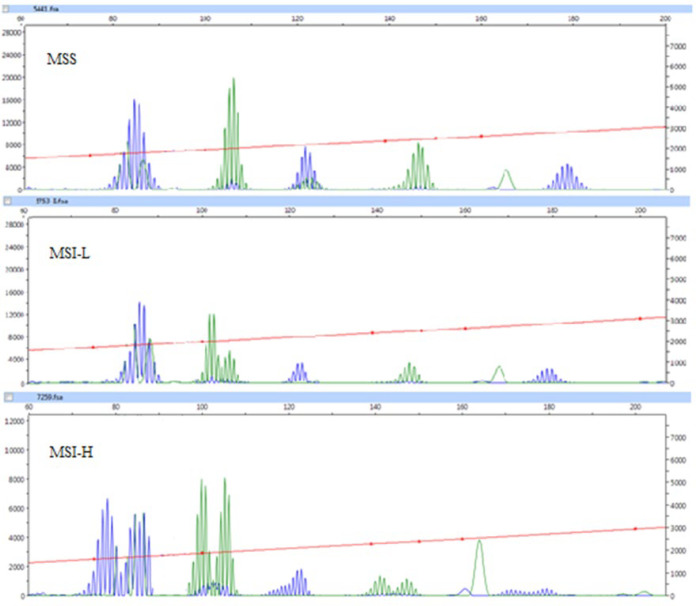
Microsatellite analysis. Capillary electrophoresis data show stability (MSS) and instability (MSI-L & MSI-H) of STR markers

After receiving valuable melt curves, all the products were sent to Macrogene Company for fragment analysis. The data of capillary electrophoresis were analyzed by Peak scanner software version 1 (Applied Biosystems, CA, USA) (Figure 2). The stability and instability of the markers were defined in three categories at MSS, MSI-L, and MSI-H. 

## Results

**Clinico-Pathological Features of Colorectal Adenocarcinomas**

The patients’ age ranged from 24-88 years (54.8±16.6). Among patients, 14 (41%) were female and 20 (59%) were male. The major location of tumor was rectosigmoid (32.3 %). The cancers were located in the right colon (52 %), the left colon (41%), and the transverse colon (5.8%). The tumoral TNM stage grouping was as follows: I (11.4 %), II (73.5%), III (14.7%), and IV (0%). Sixteen (47%) tumors were well differentiated, 5 (14%) were moderately differentiated, and 13 (38.2%) were poorly differentiated, 30 (88.6%) of cases were non-mucinous, and 4 (11.4%) were of mucinous type. Four tumors (11.4%) showed heavy infiltration of lymphocytes (medullary type). All these data are summarized in Table 2.

**IHC Analysis**

IHC slides were evaluated based on the positive normal colon mucosa as positive control of the antibody. The frequencies of abnormal results of MLH1, MSH2, MSH6, and PMS2 were 11.7, 2.9, 5.8 and 17.6 %, respectively (Table 3). At the same tumor, 4 cases were abnormal both for MLH1 and PMS2 and one case was abnormal for both MLH1 and MSH6. One case showed abnormal MLH1, MSH6 and PMS2 (Table 4). In our set based on the IHC results, 76.5% of cases showed MSS phenotype, whereas 23.5% of cases were instable (Table 2).

**Table 2:** Demographic findings in 35 colon cancer cases suspicious to microsatellite instability

Characteristics	Findings	
**Number**	34 cases	
**Female/Male**	14/20 (41%/59%)	
**Age**	24-88 (54.79±16.62)	
**Location**	Cecum	6(17.6%)
	Cecum and Ascending colon	1(2.9%)
	Ascending colon	10 (29%)
	Hepatic flexure	1(2.9%)
	Transverse colon	2(5.8%)
	Splenic Flexure	1(2.9%)
	Descending colon	2(5.8%)
	Sigmoid	6(17.6%)
	Recto-sigmoid	3 (8.8%)
	rectum	2 (5.8%)
**Histopathologic picture**	Well differentiated adenocarcinoma	16 (47%)
	Moderately differentiated adenocarcinoma	5 (14.7%)
	Poorly differentiated adenocarcinoma	13 (38.2%)
	Mucinous production	4(11.4%)
	Medullary type	4(11.4%)
**Tumor size**	3-13 cm (5 ±3.2)	
**TNM staging**	I	4 (11.4%)
	IIA	24(68.5%)
	IIB	0
	IIC	1 (2.9%)
	IIIA	1(2.9%)
	IIIB	3 (8.8%)
	IIIC	1(2.9%)
	IV	0
**Familial colon cancer history**	11(32.3%)	
**MSI ** ^1^ **-High (molecular)**	12(35.2%)	IHC: 8(23.5%)
**MSI-Low (molecular)**	9	
**MSS** ^ 2 ^ **(molecular)**	13(25.7%)	IHC: 26 (76.5%)

MSI=Microsatellite instability MSS= Microsatellite stability

**Table 3:** Microsatellite instability according to individual marker by immunohistochemistry

Markers	Percentage of loss
**MLH1**	6 (17.6%)
**MSH2**	1 (2.9%)
**MSH6**	2 (5.8%)
**PMS2**	6 (17.6%)

**Table 4:** Microsatellite instability according to individual marker by immunohistochemistry

Markers	Percentage of loss
MLH1+PMS2	4(11.7%)
MLH1+MSH6	1 (2.9%)
MLH1+MSH6+PMS2	1(2.9%)
MSH2 alone	1(2.9%)
PMS2 alone	1(2.9%)

**Table 5:** Microsatellite instability and mutation according to individual gene by DNA sequencing

Gene	Percentage of Mutant
NR21	10(29.4%)
NR24	5(14.7%)
NR27	16(47%)
BAT 25	9(26.4%)
BAT 26	4(11.7%)

**Table 6.** Comparison of different clinicopathologic characteristics between MSS and MSI cases

		MSS	MSI-L	MSI-H	MSI	P-value
**Sex**	**F**	**10**	**2**	**2**	4	0.00086
	**M**	**3**	**7**	**10**	17	
**Age**	<50	3	4	5	9	0.24
	>50	10	5	7	12	
**Location**	**Right Colon**	**6**	**4**	**8**	12	0.53
	**Transverse colon**	**0**	**1**	**1**	2	0.25
	**Left Colon**	**7**	**4**	**3**	7	0.23
**Size (cm)**	<5 cm	8	6	2	8	0.18
	>5 cm	5	3	10	13	
**TNM staging**	I	1	2	1	3	0.56
	IIA	10	5	10	15	0.7
	IIB	0	0	0	0	-
	IIC	1	0	0	0	0.19
	IIIA	0	0	0	1	0.42
	IIIB	1	1	1	2	0.85
	IIIC	0	1	0	1	0.42
	IV	0	0	0	0	-
**Histologic type**	**Well differentiated adenocarcinoma**	**8**	**2**	**4**	8	0.18
	**Moderately differentiated adenocarcinoma**	**1**	**4**	**0**	4	0.36
	**Poorly differentiated adenocarcinoma**	**4**	**3**	**8**	7	0.87
	**Mucinous production**	**0**	**0**	**4**	4	0.09
	**Medullary type**	**0**	**0**	**4**	4	0.031
**Familial Colon Cancer**		0	4	7	11	0.0003
**Second cancer**		5	3	3	6	0.5

**Frequency of MSI Status Based on DNA Sequencing**

Using molecular investigation and DNA sequencing, based on the Bethesda criteria, 12 cases (35.2%) were MSI-High and 9 cases (25.7%) showed MSI-low pattern. Table 5 shows each individual abnormal genotype. 

Three cases showed instability in 4 analyzed markers, 4 in three markers, and 5 in two markers. For the remaining 13 cases, the tumors were MSS.

All the 34 cases in this study were clinically or pathologically suspected to MSI based on the Revised Bethesda Guidelines for the testing colorectal tumors (9).

Indeed, among these MSI patients, 13 cases showed conservation of the expression of four MMR proteins by immunohistochemistry. Our results based on the molecular study (gold standard) showed a specificity of 100% and low sensitivity of 38% for the immunohistochemical analysis of microsatellite instability (Table 2).

**Association Between Microsatellite Instability and Clinicopathological Features**

The association between MSI and clinicopathological characteristics of the tumors is presented in Table 6. Microsatellite instability was significantly more common in male patients, medullary histopathology and familial colorectal cancer with P-value of 0.003, 0.03, and 0.008, respectively. 

## Discussion

MSI pathway is involved in the pathogenesis of about 10–15% of sporadic colon cancers and majority of the cases with HNPCC syndrome. MSI is mutation and somatic inactivation of DNA mismatch repair (MMR) genes, including hMLH1 (human MutL homolog 1), hMSH2 (human MutS homologue 2), hMSH6, and hPMS2 (human post-meiotic segregation 2) (1). Clinically, CRCs are classified as MSI-H (high level MSI i.e. colon cancers with instability at around 30–40% of markers), MSI-L (low level MSI i.e. colon cancers with instability at less than 30–40% of markers), and MSS (microsatellite stable i.e. colon cancers with no apparent instability) based on the extent of MSI. The cause of this inactivation is the epigenetic silencing through promoter hyper-methylation (10).

According to our findings, MSI tumors were more common in male patients (*P*<0.05). There are controversial reports in the literature, some reported equal incidence in men and women and some has reported male predominance such as our study (11), (12).

In our study, no statistically significant age difference was observed between MSI-H and MSI-L colorectal cancers (*P*>0.05). In most of the previous studies, MSI cases have been more common in the ages below 50 from European or African populations (12, 13). However, this has not been confirmed in other studies from other geographic areas such as Greece (14). In our study, MSI tumors were more common in the right colon (although it has not been statistically significant). In majority of the previous reports, MSI colorectal cancers were more common in the right colon (11, 14).

In this study, we tried to compare histopathological and immunohistochemical findings of MSI and MSS colorectal cancers. In our 34 patients with colorectal cancer, MSI tumors were more commonly located in the right colon and tended to produce mucin and showed heavy lymphocytic infiltration. Although the differences were not significant (*P*>0.05). This has also been proved in the previous reports (7, 13).

Most of our cases were in the stage II both in MSI and MSS colorectal cancer groups, thus, there was no difference between two groups according to the stage, but in most of the previous reports, MSS tumors were presented in higher stage (11, 14).

Our results showed immunohistochemistry as a very specific method for detection of microsatellite instability, but the sensitivity was low. This has been confirmed in a few other studies as well (15).

## Conclusion

In our population from South of Iran, MSI colorectal cancer tends to be more common in male patients of all ages, with a tendency to occur in the right colon and production of mucin with infiltration of many lymphocytes. 

Considering immunohistochemistry as the sole method in the diagnosis of microsatellite instability and in comparison with molecular studies using DNA sequencing, immunohistochemical procedure are associated with missing a lot of MSI tumors due to the low sensitivity (presence of high rate of false negative results). Therefore, we recommend perfor-ming molecular studies and DNA sequencing in the cases with clinical suspicion for MSI and negative immunohistochemical results. 
